# The association of visceral pleural invasion with skip N2 metastasis on clinical stage IA NSCLC

**DOI:** 10.1016/j.clinsp.2024.100334

**Published:** 2024-03-14

**Authors:** Fabio Minamoto, Pedro Araújo, Paula D'Ambrosio, Alberto Dela Vega, Leticia Lauricella, Paulo Pêgo-Fernandes, Ricardo Terra

**Affiliations:** a Instituto do Cancer do Estado de São Paulo (ICESP), Hospital das Clínicas, Faculdade de Medicina, Universidade de São Paulo (HCFMUSP), São Paulo, SP, Brazil; b Instituto do Coração (INCOR), Hospital das Clínicas, Faculdade de Medicina, Universidade de São Paulo (HCFMUSP), São Paulo, SP, Brazil

**Keywords:** Lung cancer, Visceral pleura invasion, Nodal upstaging, Mediastinal staging, Skip N2 metastasis

## Abstract

**Background:**

Lung lymphatic drainage occurs mainly through a peribronchial path, but it is hypothesized that visceral pleural invasion could alter this path. This study aims to investigate the association between visceral pleural invasion, node upstaging, and N2 skip metastasis and the impact on survival in a population of patients with non-small cell lung cancer of 3 cm or smaller.

**Methods:**

We retrospectively queried our institutional database of lung cancer resection for all patients with clinical stage IA NSCLC between June 2009 and June 2022. We collected baseline characteristics and clinical and pathological staging data. Patients were classified into two groups: The non-VPI group with negative visceral pleural invasion and the VPI group with positive. The primary results analyzed were the occurrence of nodal upstaging, skip N2 metastasis and recurrence.

**Results:**

There were 320 patients analyzed. 61.3 % were women; the median age was 65.4 years. The pleural invasion occurred in 44 patients (13.7 %). VPI group had larger nodules (2.3 vs. 1.7 cm; *p* < 0.0001), higher 18F-FDG uptake (7.4 vs. 3.4; *p* < 0.0001), and lymph-vascular invasion (35.7 % vs. 13.5 %, *p* = 0.001). Also, the VPI group had more nodal disease (25.6 % vs. 8.7 %; *p* = 0.001) and skip N2 metastasis (9.3 % vs. 1.8 %; *p* = 0.006). VPI was a statistically independent factor for skip N2 metastasis. Recurrence occurred in 17.2 % of the population. 5-year disease-free and overall survival were worse in the VPI group.

**Conclusions:**

The visceral pleural invasion was an independent factor associated with N2 skip metastasis and had worse disease-free and overall survival.

## Highlights

• Visceral Pleural Invasion (VPI) in lung cancer is associated with skip N2 metastasis.
• Nodal upstaging is linked to lymph-vascular invasion.
• VPI impacts disease-free and overall survival rates.
• Consideration of VPI in treatment decisions is crucial for improved patient outcomes.

## Introduction

As the World Health Organization reported in 2020, cancer is the leading global cause of mortality, with lung cancer emerging as the second most prevalent diagnosis and the leading cause of cancer-related death.^1^ Early detection of lung cancer presents a formidable challenge, given that symptoms often remain latent until the disease reaches advanced stages, and only a limited number of nations have implemented comprehensive lung cancer screening initiatives. The standard of care for localized lung cancer remains lung resection, boasting a 5-year survival rate ranging from 77 % to 92 % for stage IA cases.^2^

In the pathological examination, visceral pleural invasion status assumes critical significance, particularly in peripheral tumors. This intricate feature wields a profound influence on prognosis, as tumors bearing this hallmark are categorized as pT2a, irrespective of the size of the invasive component (up to 3 cm), and consequently, they fall under stage IB disease, with a 5-year survival rate of 66 %.^2^

While most lung lymphatic drainage follows the peribronchial route, an intriguing aspect is that approximately 9.5 % to 21.4 % of drainage occurs directly through the visceral pleura to the mediastinal lymph nodes.^3^,^4^ The lymphatic system of the lung is divided into deep and superficial lymphatic vessels. The first ones follow the peribronchial pathway, and the lymphatic drainage takes place to the N1 lymph nodes and subsequently to the N2 stations. The superficial ones consist of capillary nets of subpleural lymphatic vessels, and some drain directly to the mediastinum. The impact of tumor pleural invasion on these direct lymphatic pathways and its potential implications for lymph node metastasis remains uncharted. Skip N2 metastasis is a well-known phenomenon in lung cancer, and many authors associate its occurrence with the dissemination of tumor cells through the superficial lymphatic pathways.

This study aims to elucidate whether tumoral visceral pleural invasion influences lymphatic pathways, potentially leading to increased lymph node upstaging and the emergence of skip N2 metastases, ultimately culminating in diminished patient survival rates.

## Patients and methods

### Ethical statement

This was a single-center retrospective study performed at the Instituto do Cancer do Estado de São Paulo (ICESP), Hospital das Clínicas HCFMUSP, Faculdade de Medicina, Universidade de Sao Paulo. The Institutional Review Board approved the study (CAPPesq HCFMUSP ‒ 46,425,521.0.0000.0068). Since our study was retrospective and analyzed anonymously, the committee waived the need for consent.

### Data availability statement

The data underlying this article were accessed from the Brazilian Registry of Lung Cancer database. The data generated in this research will be shared at reasonable request to the corresponding author.

### Data source and patient selection

We conducted a retrospective review of a comprehensive multi-institutional database, specifically the Brazilian Registry of Lung Cancer, hosted on RedCap. This review included patients who had undergone surgical resection for non-small cell lung cancer (NSCLC) and had received systematic lymph node dissection, and the data collection period spanned from June 2009 to June 2022.

Our inclusion criteria comprised patients with NSCLC tumors characterized by a solid component of ≤3 cm in diameter, as measured from chest CT scans and the absence of nodal and distant metastases confirmed by PET-CT scans, ultimately classified as clinical stage IA according to the 8th edition of the Lung Cancer Staging Manual. These patients had undergone lobectomy, pneumonectomy, or segmentectomy, accompanied by systematic lymph node dissection at the Hospital das Clinicas ‒ FMUSP. Patients who did not meet the criteria were excluded from the study. Specifically, tumors not classified as T1 by criteria other than size, small cell lung cancer, secondary lung neoplasms, patients younger than 18, patients who did not undergo lymphadenectomy, and instances with incomplete data.

The dataset contained fundamental demographic information, including age at surgery, gender, comorbidities, smoking history, BMI (Body Mass Index), standard uptake value from PET scans, surgical approach, and the type of lung resection. Additionally, we collected postoperative pathological data on tumor size, histology, tumor grade, lymph node involvement, lymph-vascular invasion, and visceral pleural involvement. Furthermore, we monitored time-dependent variables such as recurrence and mortality during the study period. All patients underwent postoperative assessments, including chest radiographs, chest CT scans, and PET scans when clinically indicated. Pathologic diagnosis was sought to confirm the condition in cases of suspected recurrence.

For our analysis, patients were categorized into two groups based on the presence or absence of Visceral Pleural Involvement (VPI), as defined in the pathological reports. Moreover, we identified cases of Skip N2 metastasis (SN2), characterized by mediastinal lymph node involvement (pN2) without concurrent hilar lymph node involvement (pN1). Tumor grade was further classified as either well-differentiated or non-well-differentiated.

### Statistical analysis

We presented our data using appropriate statistical methods: the median and interquartile range (IQR) for non-parametric continuous variables and numbers with corresponding percentages ( %) for categorical variables. To assess the normality of continuous variables, we conducted the Shapiro-Wilk test. We employed the Mann-Whitney non-parametric *U test* to compare continuously distributed data between groups, while Fisher's exact test was utilized for categorical data comparisons between groups.

To further explore our analysis, we conducted a multivariable logistic regression analysis to identify factors significantly correlated with visceral pleural involvement, upstaging, and skip N2 metastasis. Additionally, we employed the log-rank test and Cox regression for time-dependent analysis.

All statistical analyses were conducted using Stata 14.0 (IBM, Armonk, NY, USA), with statistical significance defined as a two-sided p-value of < 0.05.

## Results

### Study population

Between June 2009 to June 2022, a total of 320 subjects underwent lung resection for primary lung cancer with nodules of 3 cm or smaller, clinical stage IA. Most patients were women (61.6 %), had a positive smoking history (70.6 %), and the median age at surgery was 65.4 years. The baseline characteristics of this population are shown in Table 1. 53 patients (15.7 %) had subsolid lung nodules. The median invasive component size of the cancer was 1.8 cm (IQR = 1.2 cm). The nodule's median uptake value on the PET scan was 4.1 (IQR = 5.8). Most of the patients underwent lobectomy (88.0 %) for lung cancer, with Video-Assisted Thoracoscopic Surgery (VATS) as the most common approach (66.5 %). All patients had R0 microscopically margin-negative resection.

**Table 1:** Study cohort demographics and clinical characteristics (*n* = 328).

Clinicopathological features and diagnostic/management approaches		Number (patients)	Percent (%)
Age	Years (median/IQR)	65.4 / 12.3	
Gender	Male / female	123 / 197	38.4 / 61.6
Body mass index	Kg/m^2^ (median / IQR)	25.7 / 6.8	
Smoking history	Ever	224	70.7
	Never	93	29.3
Solid nodule		263	84.3
Surgical approach	Thoracotomy	74	23.1
	VATS	213	66.6
	RATS	33	10.3
Tumor size	cm (median/IQR)	1.8 / 1.2	
Tumor 18F-FDG uptake	(median/IQR)	4.1 / 5.8	
Histological subtype (%)			
Adenocarcinoma	Acinar	91	28.4
	Lepidic	34	10.6
	Papillary	27	8.4
	Solid	19	5.9
	Mucinous	14	4.4
	Micropapillary	6	1.9
Squamous cell carcinoma		41	12.8
Carcinoid tumor		50	15.6
Tumor grade (%)	G1	65	26.7
	G2	131	52.7
	G3	44	18.1
Lymph-vascular invasion		52	16.8
Visceral pleura invasion	PL0	276	86.2
	PL1	27	8.4
	PL2	17	5.3
Nodal Upstaging	N1	17	5.3
	N2	18	5.6
Skip N2 metastasis		9	2.8
Surgical approach	Thoracotomy	74	23.1
	VATS	213	66.6
	RATS	33	10.3
Lung ressection	Lobectomy	284	89.3
	Segmentectomy	34	10.7

Baseline characteristics of the study population.

Two hundred and seventy-six patients (86.2 %) had negative pleural invasion (PL0) and were categorized into the non-VPI group. 44 patients (13.8 %) had positive visceral pleural invasion and were classified into the VPI group, 27 of them (8.4 %) PL1 and 17 (5.3 %) PL2. The most common lung cancer was adenocarcinoma (67.2 %), with the acinar subtype the most frequent (28.8 %). Thirty-five patients (10.9 %) had nodal upstaging, with 5.3 % N1 and 5.6 % N2. Skip N2 metastasis occurred in 2.8 % of the cohort. The lymph-vascular invasion was found in 52 patients (16.2 %). Well-differentiated tumors represented 27.2 % of all cases.

### Group comparison

Table 2 details the difference between non-VPI and VPI groups. Patients with visceral pleural invasion had larger tumors (median size 2.3 vs. 1.7 cm, *p* < 0.0001), higher uptake of FGD on PET (median SUVmax 7.4 vs. 3.4, *p* < 0.0001), mostly non-well differentiated tumors (90.9 % % vs. 9.1 %, *p* = 0.011), and more lymph-vascular invasion (35.7 vs. 12.8, *p* = 0.002). On multivariable analysis, only the tumor size was statistically associated with the occurrence of visceral pleural invasion (OR = 2.8, *p* = 0.017). Neither group had a statistical difference regarding the lung cancer subtype and subsolid nodules. Moreover, group 2 had nodal upstaging (25.6 % % vs. 8.7 %, *p* = 0.001), more lymphatic N2 skip metastasis (9.3 % % vs. 1.8 %, *p* = 0.006), higher rate of recurrence (*p* = 0.08) and higher overall mortality (*p* = 0.02).

**Table 2:** Group comparison ajusted by VPI.

	Negative pleural involvement (Non-VPI group)	Positive pleural involvement (VPI group)	Univariable analysis (p)	Multivariable analysis (p)
Median size (cm) – IQR	1.7 (1.05)	2.3 (0.9)	*p* < 0.0001	0.017
Median SUV max – IQR	3.4 (5.1)	7.4 (6.05)	*p* < 0.0001	0.36
Solid nodule (%)	82.4	90.0	0.28	
Tumor grade (%)			0.011	0.93
Well-differentiated	30.2	9.1		
Non-well-differentiated	69.8	90.9		
Subtype (%)			0.49	
1. Adenocarcinoma				
Acinar	27.1	34.9		
Lepidic	10.5	11.6		
Papillary	8.7	7.0		
Solid	5.4	9.3		
Mucinous	4.7	2.3		
Micropapillary	1.4	4.6		
2. Squamous cell carcinoma	12.7	13.9		
3.Typical carcinoid tumor	13.7	2.3		
4. Atypical carcinoid tumor	3.6	2.3		
Lymp-vasc invasion	13.5	35.7	0.001	0.99
Lung ressection			0.22	
Lobectomy	87.2	97.7		
Segmentectomy	12.0	2.8		
Nodal upstaging	8.7	25.6	0.001	0.28
Skip N2 metastasis	1.8	9.3	0.006	0.52

Group comparison-adjusted by visceral pleural invasion, with univariable and multivariable results. Tumor size was an independent factor associated with VPI. OR, Odds Ratio.

Nodal upstaging occurred in 10.9 % of our population; it was more frequent in the VPI group (*p* = 0.003), in solid tumors (12.5 % % vs. 2.0 %, *p* = 0.025), and was associated with lymph-vascular invasion (26.9 % % vs. 7.0 %, *p* < 0.0001), non-well differentiated tumors, larger nodules and with higher 18F-FDG uptake (Table 3). On multivariable analysis, only lymph-vascular invasion (OR = 2.2, *p* = 0.027) had a statistically relevant association with nodal upstaging. VPI was not associated with upstaging on multivariable analysis. There were no differences in nodal upstaging comparing the cancer type, surgical approach (thoracotomy, VATS, and RATS), or the lung resection performed.

**Table 3:** Nodal upstaging analysis.

	Positive lymph node upstaging	Univariable analysis (P)	Multivariable analysis (P)
Median size (cm) – IQR	2.2 (1.1)	0.01	0.34
Median SUV max – IQR	8.3 (8.7)	0.001	0.13
Solid nodule (%)	97.1	0.30	
Visceral pleura invasion (%)		0.001	0.12
Positive	25.6		
Negative	8.7		
Lymph-vasc invasion (%)		*p* < 0.001	0.01
Positive	26.9		
Negative	7.0		
Tumor grade (%)		0.005	0.43
Well-differentiated	3.8		
Non-well-differentiated	96.2		
Lung resection (%)		0.15	
Lobectomy	12.5		
Segmentectomy	0		
Surgical approach (%)		0.36	
Thoracotomy	14.8		
VATS	10.3		
RATS	6.1		

Nodal upstaging analysis. This table shows the variables associated with nodal upstaging and the statistical analysis.

Skip N2 metastasis occurred in 2.81 % of our patients; it had a positive association with visceral pleural invasion (9.3 % % vs. 1.8 %, *p* = 0.006) and lymph-vascular invasion (7.7 % % vs. 1.9 %, *p* = 0.025). Tumor size, FDG uptake, and tumor grade were not associated. On multivariable analysis, only VPI was a statistically independent factor associated with it (OR = 4.4, *p* = 0.03).

**Fig. 1:**
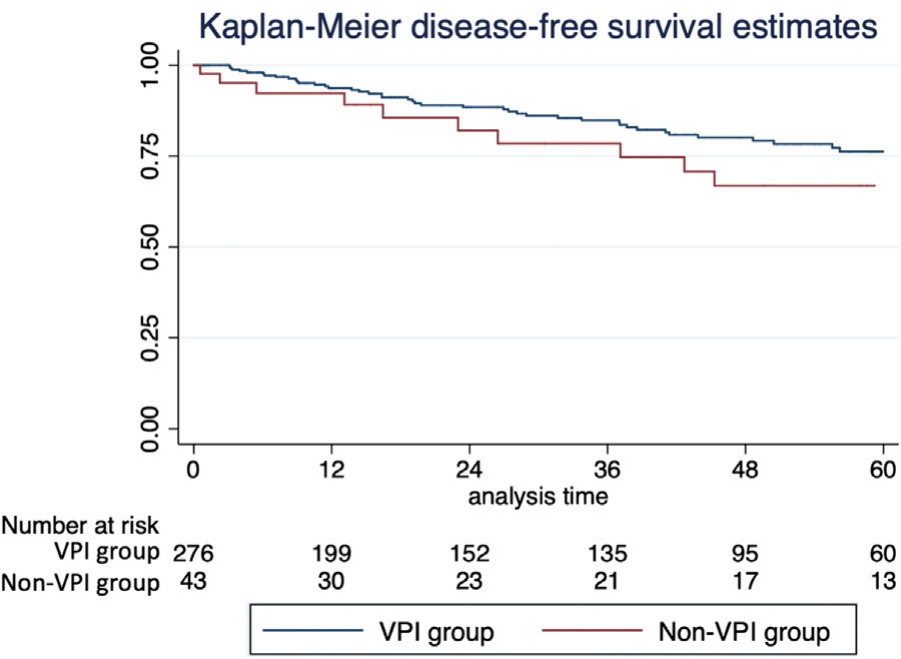
Kaplan – Meier curves with disease-free survival (A) and overall (B) estimates. Group 1 with negative visceral pleura invasion. Group 2 with positive visceral pleura invasion.

Fifty-five patients (17.2 %) had a cancer recurrence, most in the ipsilateral lung, in another lobe. On univariable survival analysis, VPI impacted disease-free survival, with lower estimated 5-year survival (66.8 % % vs. 76.2 %, *p* = 0.08), and overall survival, with lower estimated 5-year survival (69.4 % % vs. 79.4 %, *p* = 0.025); see the Kaplan-Meier curves (Fig. 1). However, VPI was not statistically associated with recurrence and death on multivariable analysis on Cox regression.

## Discussion

The eighth edition of the TNM staging manual for NSCLC classifies tumors with visceral pleural invasion as T2a, even if they are less than 3 cm in size. Visceral pleural invasion of lung cancer is an important prognostic factor.^2^^,^^5^, ^6^, ^7^ It is typically identified by postoperative pathological testing because it may be challenging to diagnose by preoperative imaging or intraoperative macroscopic findings. It is not common in small peripheral nodules, and some studies report VPI in 9.7 %‒13.8 % of patients with operated tumors with ≤ 2 cm.^8^,^9^ Similarly, our cohort found an incidence of 13.8 % of VPI; however, we included lesions up to 3 cm, too, so we could group all clinical T1 patients with a more significant number of subjects and outcomes of interest, such as nodal upstaging and skip N2 metastasis.

Some studies found that patients with visceral pleural invasion are more likely to develop lymph node metastases,^6^,^9^ regardless of size. Nodal upstaging was defined as a lack of concordance between clinical and pathological staging at the N descriptor. The subset of patients with clinical stage I tumors is expected to have a relatively low number of upstaging since we consider it an initial disease. Wilson et al. published a retrospective cohort with 302 patients to assess the prevalence of upstaging during robotic resection. They had 78.5 % clinical stage IA NSCLC with 10.9 % pathological nodal upstaging and concluded that robotic surgery had a similar performance on nodal dissection to the thoracotomy approach and was superior to VATS.^10^ Our cohort had a rate of 10.8 % of pathological nodal upstaging, with equal rates of N1 and N2 disease associated with lymph-vascular invasion. This pathological feature is not included in the T descriptor of the NSCLC staging system, although some studies indicate it as an independent prognostic factor. Also, the association between upstaging and higher standard uptake of 18F-FDG was significant, which may be explained by more aggressive tumors with higher cell turnover and consumption. The surgical approach and VPI were not relevant to our multivariable statistical analysis.

The fact that VPI (Visceral Pleural Invasion) was not associated with nodal upstaging, involving both N1 and N2 pathological disease, but specifically with N2 skip metastasis, yields significant insights into distinct lymphatic drainage pathways among these subject groups. Skip metastasis, where non-small cell lung cancer directly invades mediastinal lymph nodes without involving hilar lymph nodes, is well-documented in medical literature, occurring in approximately 13.8 % to 37.8 % of cases.^11^ Visceral pleural invasion disrupts the usual lymphatic drainage patterns, directing neoplastic cells along a direct route to the mediastinum bypassing pulmonary lymph nodes. This phenomenon has been substantiated in cadaveric studies, such as the one conducted by Riquet et al.,^3^ which involved 260 subjects receiving subpleural dye injections. Their study revealed a 23.6 % incidence of direct passages to mediastinal nodes. In our cohort, while we observed a skip N2 metastasis rate of 2.8 %, it is essential to note that our study focused exclusively on pathological T1 to T2a tumors. Our findings confirm the independent association between visceral pleural invasion and skip N2 metastasis, aligning with Riquet's observations. Similarly, Gorai's research^12^ also corroborates this association in a similar group of NSCLC (Non-Small Cell Lung Cancer) patients. In our study and Gorai's, patients with clinical stage IA disease exhibited this connection between VPI and skip N2 metastases. While we found that tumor size was not significantly linked to skip N2 metastases, it did show a strong correlation with the occurrence of VPI. The relationship between these variables suggests that both contribute to the phenomenon.

Recently, the randomized trial JCOG0802 indicates that segmentectomy should be the standard surgical procedure for small, < 2 cm, and peripheral nodules.^9^ However, the aspects of the T descriptor regarding the pleural invasion are not discussed. The question remains, do patients with ≤ 2 cm nodules and VPI who have undergone sub-lobar resection have a similar outcome to those who have undergone lobectomy? Or should the JCOG0802 recommendation exclude patients with suspected pleural invasion? Our evidence suggests that these patients have a different evolution, so we must define the treatment decision according to the clinical staging T2a, which recommends lobectomy as standard surgery. How to assess visceral pleural invasion is the next ponderation. It is not easy to define it on radiological exams since it is a microscopic feature. The advances in operative technologies, such as the robotic platform, enhance the quality of intra-operative images and new features. Takizawa et al. evaluated the autofluorescence mode of video thoracoscopy for intraoperative diagnosis of visceral pleural invasion. Twenty-five cases of patients who underwent VATS were selected, and eight independent thoracic surgeons evaluated the intraoperative images in white light and autofluorescence mode to diagnose VPI.^13^ They concluded that autofluorescence mode improved the diagnosis of this feature during surgery.

Huang et al. conducted a meta-analysis with 22 studies that demonstrate the association of VPI with higher rates of death and recurrence in stage I tumors.^14^ In addition, the 5-year survival of pT1 varies from 92 %‒81 %, while pT2a is 74 %, according to Rami-Porta et al.^15^ Our survival analysis corroborates it as demonstrated on Kaplan-Meier curves with worse overall and disease-free survival; however, it was not seen on multivariable analysis, which suggests another factor may impact these results. Nitadori et al. demonstrated in 2013 that VPI was not associated with recurrence in tumors ≤ 2 cm but was an independent prognostic factor in the subgroup with 2 to 3 cm.^16^

It is essential to note the biases and limitations of this study. Patient selection and time trend biases regarding the diagnosis of cancer recurrence and follow-up may be unavoidable in a retrospective study carried out at a single institution. Moreover, the small number of patients should be mentioned as a potential cause of statistical power loss. It also makes it unfeasible to perform a propensity score match analysis, which could restrict the inference to the matched cohort and potentially limit generalizability. Tumor grade and STAS might have impacted the results; however, both variables were excluded due to incomplete data on the older subjects. The new classifications were included in the most recent series and were not available back then. Since a significant part of our cohort had the surgery over 5 years ago, and we cannot retrieve the biological specimen of most of these cases, we decided not to carry on with the re-study of these samples by a pathologist.

## Conclusion

In summary, our study highlights the significance of Visceral Pleural Invasion (VPI) in lung cancer. VPI is closely linked to skip N2 metastasis and poorer survival rates. Nodal upstaging is also associated with lymph-vascular invasion. These findings emphasize the importance of considering VPI in treatment decisions and prognosis, especially for smaller tumors. However, our study has limitations, including its retrospective nature and small sample size. Future research should delve further into VPI, its detection methods, and its impact on treatment, aiming to enhance lung cancer management and patient outcomes.

## Declaration of Generative AI and AI- assisted technologies in the writing process

During the preparation of this work, the author(s) did not use Generative AI and AI-assisted technologies in the writing process.

## Funding

This study had no funding.

## CRediT authorship contribution statement

**Fabio Minamoto:** Conceptualization, Data curation, Formal analysis, Investigation, Methodology, Project administration, Writing – original draft, Writing – review & editing. **Pedro Araújo:** Conceptualization, Formal analysis, Investigation, Methodology, Writing – review & editing. **Paula D'Ambrosio:** Methodology, Writing – review & editing. **Alberto Dela Vega:** Conceptualization, Data curation, Writing – review & editing. **Leticia Lauricella:** Data curation, Formal analysis, Project administration, Writing – review & editing. **Paulo Pêgo-Fernandes:** Formal analysis, Project administration, Writing – review & editing. **Ricardo Terra:** Conceptualization, Formal analysis, Data curation, Supervision, Writing – review & editing.

## Declaration of competing interest

The authors declare no conflicts of interest.
